# Molecular evolution of cytochrome C oxidase-I protein of insects living in Saudi Arabia

**DOI:** 10.1371/journal.pone.0224336

**Published:** 2019-11-04

**Authors:** Jamal S. M. Sabir, Samar Rabah, Haitham Yacoub, Nahid H. Hajrah, Ahmed Atef, Mohammed Al-Matary, Sherif Edris, Mona G. Alharbi, Magdah Ganash, Jazem Mahyoub, Rashad R. Al-Hindi, Khalid M. Al-Ghamdi, Neil Hall, Ahmed Bahieldin, Majid R. Kamli, Irfan A. Rather

**Affiliations:** 1 Department of Biological Sciences, Faculty of Science, King Abdulaziz University (KAU), Jeddah, Saudi Arabia; 2 Department of Biological Sciences, Faculty of Science, University of Jeddah, Dahaban, Saudi Arabia; 3 Department of Genetics, Faculty of Agriculture, Ain Shams University, Cairo, Egypt; 4 Princess Al-Jawhara Al-Brahim Centre of Excellence in Research of Hereditary Disorders (PACER-HD), Faculty of Medicine, King Abdulaziz University (KAU), Jeddah, Saudi Arabia; 5 The Genome Analysis Center, Norwich Research Park, Norwich, United Kingdom; Dongguk University, REPUBLIC OF KOREA

## Abstract

The study underpins barcode characterization of insect species collected from Saudi Arabia and explored functional constraints during evolution at the DNA and protein levels to expect the possible mechanisms of protein evolution in insects. Codon structure designated AT-biased insect barcode of the cytochrome C oxidase I (COI). In addition, the predicted 3D structure of COI protein indicated tyrosine in close proximity with the heme ligand, depicted substitution to phenylalanine in two Hymenopteran species. This change resulted in the loss of chemical bonding with the heme ligand. The estimated nucleotide substitution matrices in insect COI barcode generally showed a higher probability of transversion compared with the transition. Computations of codon-by-codon nonsynonymous substitutions in Hymenopteran and Hemipteran species indicated that almost half of the codons are under positive evolution. Nevertheless, codons of COI barcode of Coleoptera, Lepidoptera and Diptera are mostly under purifying selection. The results reinforce that codons in helices 2, 5 and 6 and those in loops 2–3 and 5–6 are mostly conserved and approach strong purifying selection. The overall results argue the possible evolutionary position of Hymenopteran species among those of other insects.

## Introduction

The fact that many insect species are difficult to be discriminated at the morphological level, as well as the huge number of cryptic species, makes the global species count uncertain [1]. The adoption of DNA-based molecular markers represents a satisfactory alternative. Since the proposal of DNA barcoding in 2003, subunit I (658 bp) of the mitochondrial cytochrome C oxidase (COX) gene (namely COI) became the most universal marker for species identification in the animal kingdom [2,3]. The recently developed Barcode Index Number (BIN) system [4] (Ratnasingham and Hebert, 2013) can act as a powerful alternative to morphological species that easily distinguishes the occurrence of diversity or possible speciation [5,6,7,8,9,10,11,12]. Technically speaking, this system complements the molecular-based approaches of species identification to strengthen and support the evolutionary analysis in insects.

Documentation of insect diversity in the Sahara-Arabian region including Saudi Arabia has recently taken place [13] as the biodiversity data available for this region is insufficient, compared with those in Canada or the US. Documentation based on the criteria of the International Union for Conservation of Nature allows recognizing invasive alien species or list threatened species. Malaise traps were successfully used in scoring richness of insect species and biodiversity surveillance in regions that are difficult-to-access [14] and/or in the absence of formal taxonomic assignments [15].

COX enzyme functionally participates in the electron transport chain by reducing oxygen and pumping protons across the inner mitochondrial membrane. The encoded protein of COI comprising 219 amino acids (AAs) consists of six polypeptide chains and a few metallic ligands. The latter includes two iron atoms bound in two heme groups, three coppers, one zinc and one magnesium [16,17]. Changes in the AA sequence within the COI region likely reduce cellular energy metabolism, especially when changes occur close to the active sites of the enzyme [18]. Purifying selection predominantly occurs for animal mitochondrial protein-coding genes, thus, results in the scarce of AA substitutions, especially in the COX genes [19,20,21]. However, evidence of nonsynonymous AA substitution supports the notion of positive selection generally in animal and specifically in class Insecta. Due to the functional constraints criterion, changes should be biased to the nonfunctioning regions of the gene supporting the claim that evolution is not neutral [20,22]. Positive selection for changes in mitochondrial proteins during evolution could be caused by lifestyle changes, and this has been observed in snakes changing their metabolic rate to complement lifestyle [21].

In this work, we introduce a model study on the COI subunit of specimens of six insect orders collected from Saudi Arabia to generate DNA barcodes and detect variation among species and orders at the AA level. Based on the latter, changes in COI protein structure at the three-dimensional model are projected. Through this model study, we gained new insights into the possible mechanisms by which COI protein evolves in insects living in Saudi Arabia.

## Materials and methods

For sample collection, a Malaise trap [23,24] (Hutcheson and Jones, 1999; Hill et al., 2005) was installed at Hada Al-Sham station, King Abdulaziz University (KAU) located in the western region (near Makkah) of Saudi Arabia (21.795^o^N, 39.711^o^E). Samples were collected on a weekly basis for four weeks (May 1–28, 2017) into 95% ethanol and stored at -20°C for further analysis. Specimens were morphologically classified down to the species level, then total DNAs were recovered from individual tissue samples according to Evans and Paulay [25]. PCR was performed to recover the COI gene fragment (650 bp) as described by Ashfaq et al. [13] and amplicons were shipped to Macrogen (South Korea) for Sanger sequencing. Recovered sequences were checked for quality and those meeting the standard criteria were further assigned to subsequent analysis. Good quality sequences were trimmed and algorithmically aligned with Clustal Omega with strict parameters and the resulting alignments were manually refined before translation. Deduced AA sequences were recovered consulting the invertebrate mitochondrial code. Samples with nucleotide deletions were excluded upon DNA multi-sequence alignment, and only in-frame AA sequences were retained. A consensus sequence of Odonata (accession no. JN294479) was used as a reference for comparison. This order is the most ancient in the hexapoda insect phenogram [26]. Binary data matrices were entered into TFPGA (version 1.3) and analyzed using qualitative routine to generate a similarity coefficient. Dissimilarity coefficients were used to construct dendrograms using unweighted pair group method with arithmetic average (UPGMA) and sequential hierarchical and nested clustering (Neighbor-Joining or NJ) routine using NTSYSpc (version 2.10, Exeter software). Phylogeny tree generated by Bybee et al. [26], was taken as a reference ancestral order for testing insect lineage and evolution.

The AA sequence of cattle (*Bos taurus*) was used as a reference [27] for detecting changes of COI protein of selected species at the 3D structure level. We recruited the bovine protein X-ray structure (Protein Data Bank ID 1OCC) as a homology model of the COI barcode region to build 3D structures of selected insect species using the I-TASSER Suite [28]. Distance measurements between AAs, on one side, and the two heme ligands referenced as 515 and 516, on the other side, were estimated using UCSF Chimera 1.13.1 [29]. Electrostatic potential of the COI protein was estimated using DeepView—Swiss-PdbViewer (v4.1) (http://www.expasy.org/spdbv)

Nucleotide substitution patterns, e.g., synonymous (S) and nonsynonymous (N), were generated using the Estimate Substitution Matrix feature in MEGA v. 6 and estimates of the numbers of S and N substitutions per site were made using the joint Maximum Likelihood reconstructions of ancestral states [30] of codon substitution and Felsenstein model [31] of nucleotide substitution. Changes in AA sequences were scored referring to the consensus sequence of Odonata.

## Results and discussion

COI barcode was analyzed at the DNA and protein levels for insects living in Saudi Arabia in order to gain new insights as to how this gene and its encoded protein evolve among different insect orders. A number of 560 samples were collected by the Malaise trap within a period of four weeks. Photographs were taken for all specimens, while the number was eventually narrowed to one photograph per species as shown in S1 Fig. DNAs were purified from these samples and the recovered mitochondrial COI gene fragments were sequenced. We projected to include only sequences with ≥ 634 nucleotides encoding all loops and helices of the COI protein. The number of deduced AAs is 211 starting at codon position 12 referring to the numbering made by Pentinsaari et al. [32]. Based on the rigid quality control criterion, the number of samples was narrowed to 175 for further analysis. The consensus DNA sequence of Odonata was included for comparison as this order has an ancient phylogenetic position being the representative of the first ancient winged insects in the hexapoda phylogeny [26]. This order has extensively been used as a reference in contemporary evolutionary genomic studies [33]. BLAST analysis indicated that a number of 30 species representing six insect orders were collected during the course of this study. Two of which belong to Hemimetabolous, e.g., Blattodea (one species) and Hemiptera (five species), while four belong to Holometabolous, e.g., Hymenoptera (six species), Coleoptera (six species), Lepidoptera (five species) and Diptera (seven species). One of the Hymenopteran species was not precisely identified at both the morphological and molecular levels, while showed the closest relationship (98%) with *Apoidea* sp.

**Fig 1:**
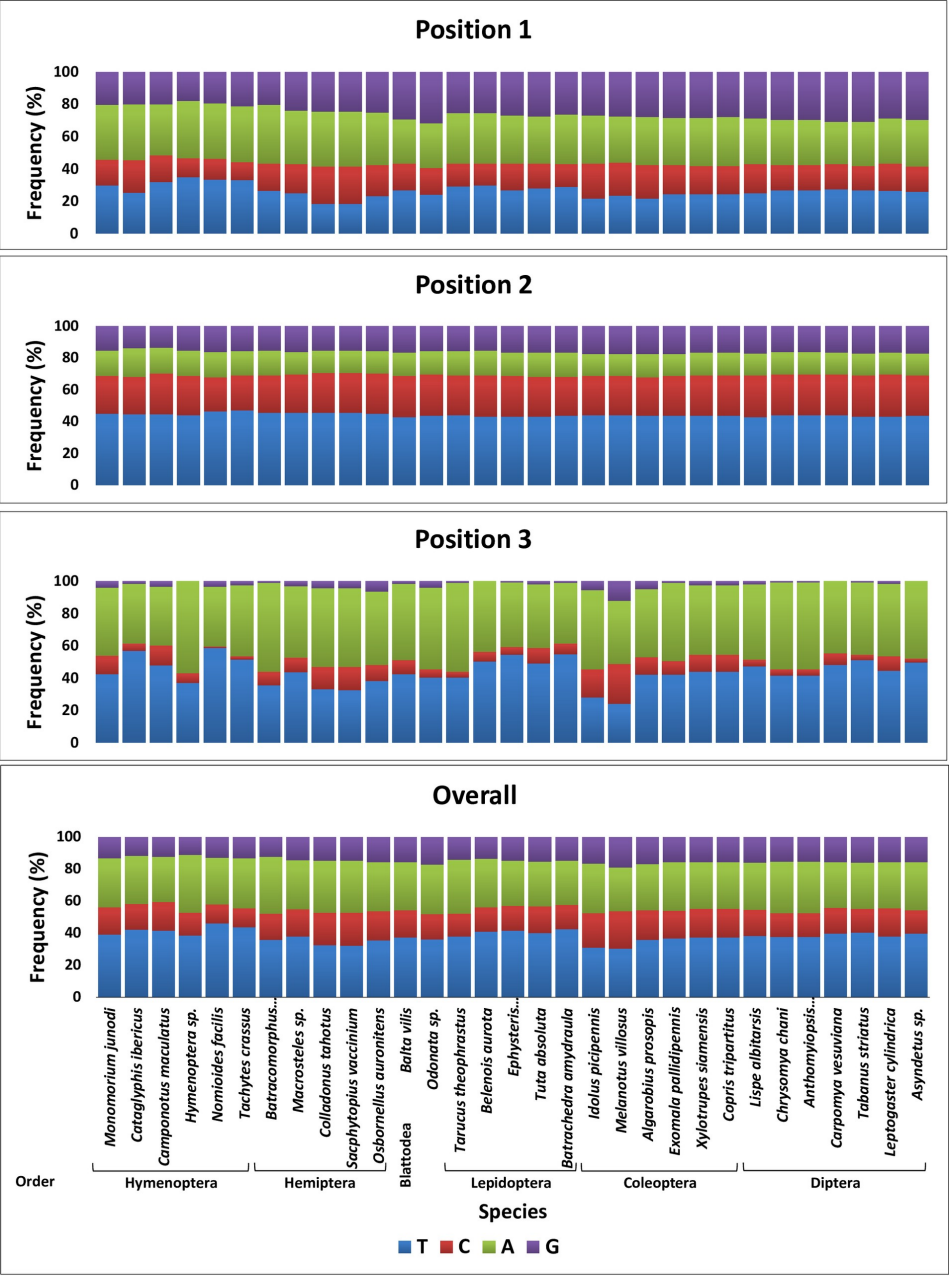
Distribution of nucleotides within codons across six insect orders. Nucleotide frequencies at each of the three codon positions in COI barcode sequence of species of six insect orders in addition to an Odonata sp. used for comparison.

Multiple DNA sequence alignment indicated that a number of 265 nucleotides out of 634 are common (conserved) in the recovered COI barcode, while the rest varied among orders and species as shown in S2 Fig. A phylogenetic tree constructed from the sequences indicated the close relationship between the species of Odonata and Blattodea as both orders are hemimetabolous (S3 Fig). Recent research indicated that Blattodea is the closest among the six orders of the present study to Odonata (Fig 1) [26]. Interestingly, a close relationship was shown between species of Hemiptera and Hymenoptera although the first is hemimetabolous, while the second is holometabolous. Species of Coleoptera were shown in close relationship with those of Diptera (S3 Fig).

As it is the case with animal mitochondrial DNA [32], the DNA COI barcode sequences analyzed for the six orders is AT-biased (e.g., AT content = ~68%) as shown in Fig 1. The AT content was lowest (~57%) for the nucleotides in the first position of all codons, while ~58% in the second position and as high as ~90% in the third position. The G nucleotide has proven to be the least degenerate among the four nucleotides in the third position. Interestingly, A nucleotide in the second position was unexpectedly low (<15%), while C was high (~25%). No G nucleotide was scored in the third position for four species namely *Hymenoptera* sp., *Belenois aurota*, *Asyndetus* sp. and *Carpomya vesuviana*. The first two species are Hymenoperan and Lepidopteran, respectively, while the other two are Dipteran. The lowest C nucleotide in the third position was scored for the two Hymenoptran species *Tachytes crassus* (<2%) and *Nomioides facilis* (<1%) (Fig 1).

**Fig 2:**
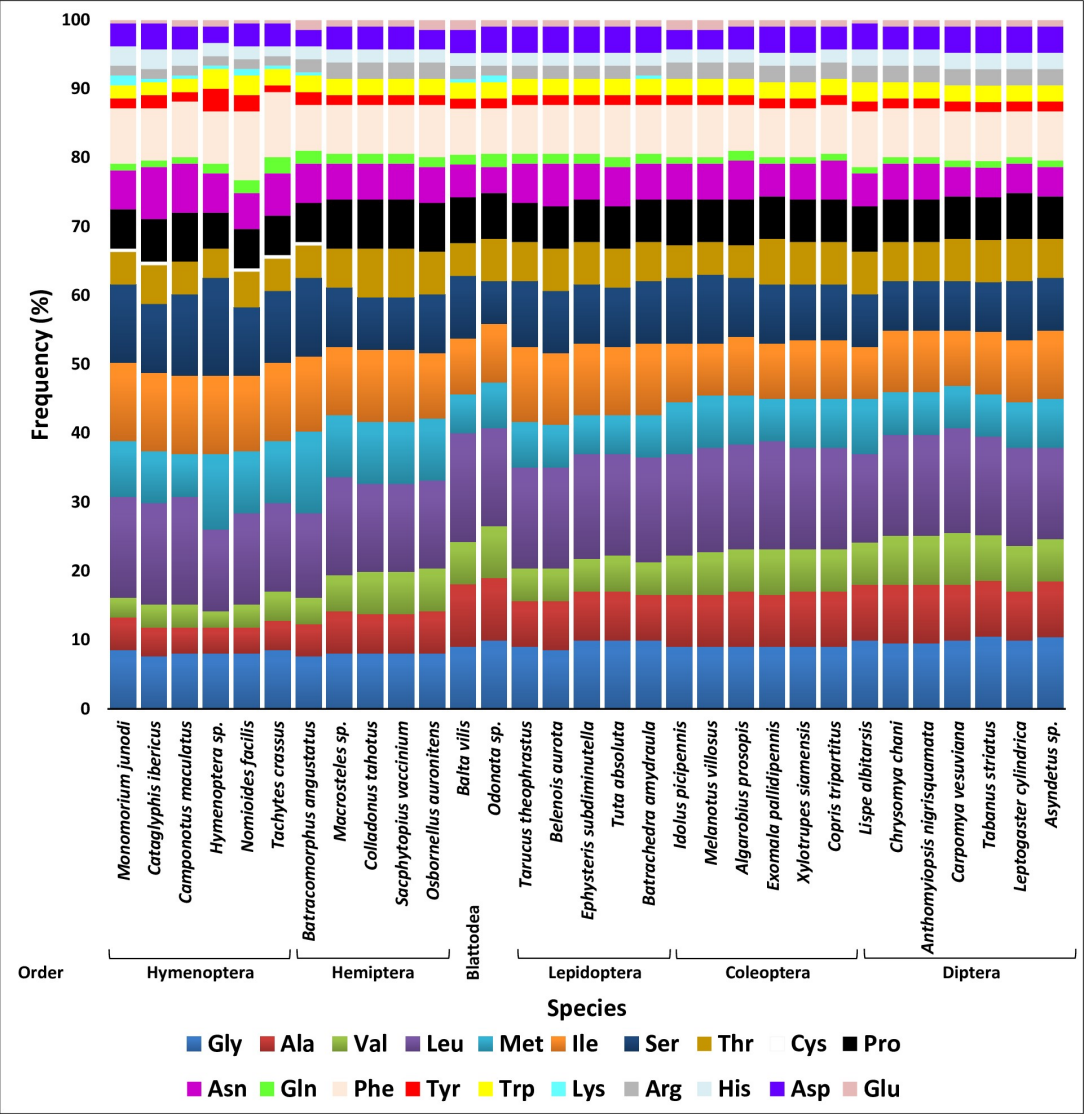
Distribution of aminoacids within the barcodes across six insect orders. Amino acid frequencies in barcode sequences of species of six insect orders in addition to an Odonata sp. used for comparison. AAs are grouped vertically based on their biochemical properties.

Amino acid sequences of COI barcode were sorted out in this study based on their chemical properties into standard groups: nonpolar aliphatic (G, A, V, L, M and I), polar uncharged (S, T, C, P, N and Q), aromatic (F, Y and W), positively charged (K, R and H) and negatively charged (D and E). The barcode sequences of different species largely encode nonpolar AAs (Fig 2). There are differences in the AA composition of the barcode sequences among orders. Alanine (A) and valine (V) are lower in barcodes of Hymenopteran species as well as of the Hemipteran species *Batracomorphus angustatus* compared with those of the other orders. On the other hand, lysine (K) is exclusively encoded by barcodes of the latter species although there are very few. Four species of the latter two orders) Hymenoptera and Hemiptera) also exclusively encode cysteine (C). The two AAs K and C do not exist in the sequence of cattle COI barcode [32]. We speculate that they appeared due to AA substitutions in several positions of the animal AA sequence of the barcode of *Batracomorphus angustatus*. Three other AAs seem to be encoded in very small amounts in few orders such as glutamine (Q), tyrosine (Y) and glutamic acid (E). Similar results were previously reached by Pentinsaari et al. [32] when studying Coleopteran and Lepidopteran species. The scarcity of these AAs in barcodes can possibly be compensated by AAs of the same chemical groups at the same position in the polypeptide chain. For example, tyrosine (Y) exists in three positions, e.g., 38, 113 and 215. The position in the middle is conserved among the six orders, while substituted to phenylalanine (F) in the other two positions. The two AAs exist in the same chemical group (aromatic). Interestingly, the five rare AAs (C, K, Q, Y, and E) are encoded by twofold degenerate codons. Cysteine in the COI region exists in four species of Hymenoptera and one species in Hemiptera although in very small amounts, while completely absent in other species of the six orders. Changes in cysteine in the barcode sequence can severely affect the secondary structure as well as the stabilization of tertiary and quaternary structures of the COI protein.

Multiple AA sequence alignment of the COI barcode of the 30 species is shown in S4 Fig. The encoded AA sequences of the DNA barcode fragment across the six orders cover 211 AAs starting from position 12 to 222 based on the numbering made by Pentinsaari et al. [32]. This portion of the barcode includes the enzymatically active part of COX mediating electron transfer from Cu to heme. Amino acid sequence variation in each position was scored in which we referred to conserved AAs by asterisks while increasing variability by reducing the number from 9 to 1 (S4 Fig). Based on the criterion followed by Pentinsaari et al. (2016), the substitution of AAs with the same chemical property at any given position is not considered to significantly influence enzyme function. This also depicts the substitution of AAs from one chemical group to the other in a position far from the enzyme ligands. However, substitutions that change the AA chemical group occur close to the enzyme ligands, may likely influence enzyme function. As indicated earlier, the secondary structure of the barcode region includes six α-helices connected by five loops (see Fig 3 in [32]). The loops encompass 62 AAs of which all the eight-plus 11 AAs of loops 1–2 and 3–4, respectively, vary among the six orders. AAs in loop 2–3 vary in three out of the eight AAs, while 10 out of 23 and five of 12 for loops 3–4 and 5–6, respectively. The loop 3–4 is important for pointing towards the heme group at the active site of the COI protein. The higher number of conserved AAs in this study was expected due to the narrow genetic distances among the six insect orders compared with those shown by Pentinsaari et al. [32] across the Metazoan barcodes. The number of the conserved AAs of the barcode sequence among the six insect order is 102 including the known 23 conserved AAs among Metazoan [32]. Overall, AA variations occurring at positions in the helices 3, 4 and 5 likely have less influence on enzyme function compared with that in the helices 1 or 2. Besides, AA variations are more pronounced in loops than in helices.

**Fig 3:**
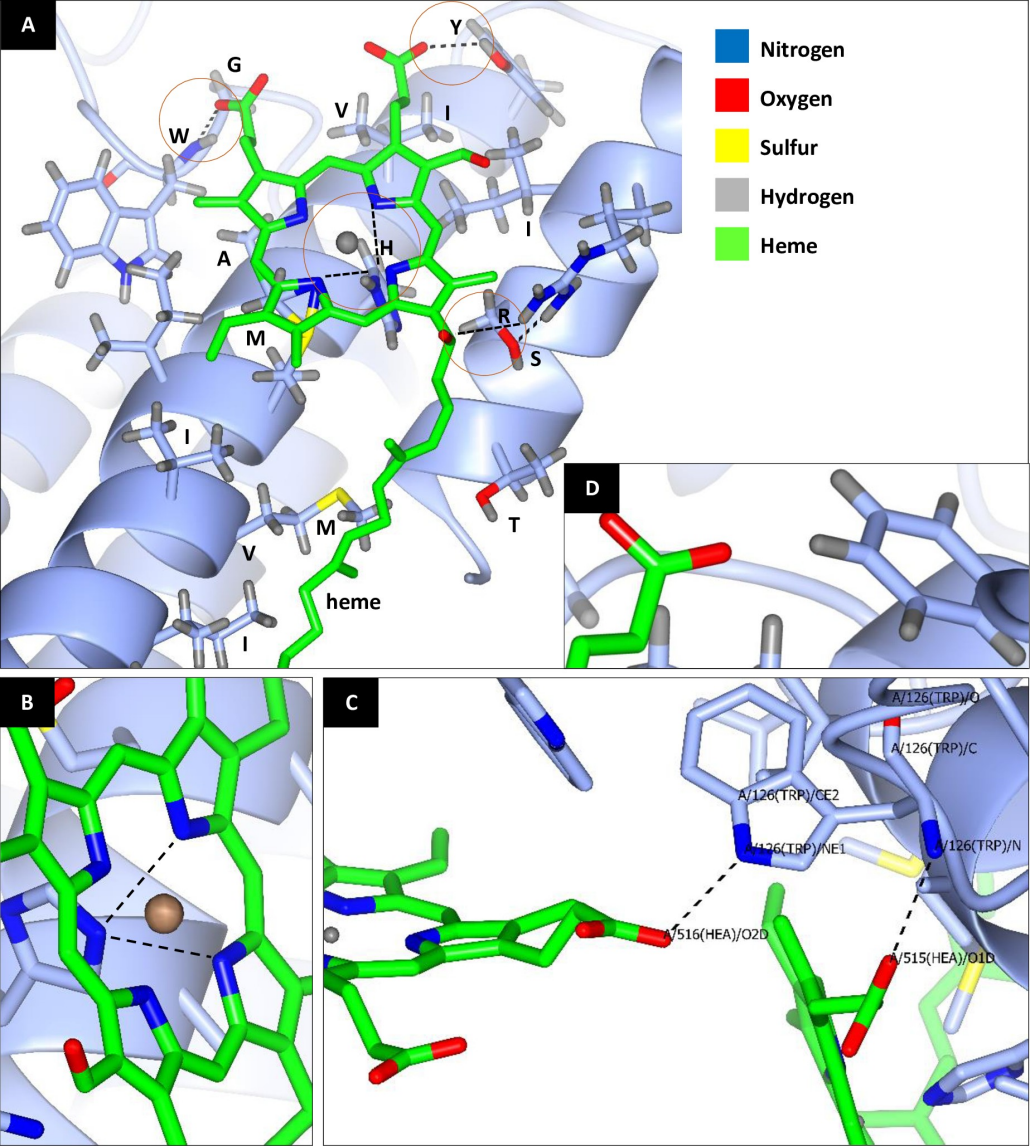
Predicted three-dimensional COI protein structure of *Batrachedra amydraula*. A model barcode of the insect consensus AA sequence in the heme 515 vicinity. **A** displays the 16 AAs that probably affect functions of the two hemes 515 and 516. These AAs are threonine (T), sulfur (S), isoleucine (I), arginine (R), tyrosine (Y), isoleucine (I), valine (V), histidine (H), alanine (A), methionine (M), isoleucine (I), methionine (M), valine (V), isoleucine (I), glycine (G) and tryptophan (W). These AAs exist at positions 15, 18, 21, 22, 38, 41, 42, 45, 46, 49, 50, 53, 54, 57, 109 and 110 of the COI polypeptide chain, respectively, following AA numbering of Pentinsaari et al. (2016). Four AAs sharing six bonds with hemes 515 and 516 are shown inside red circles. One extra bond also exists between S at position 18 and R at position 22. **B** indicates the occurrence of two bonds between H (position 45) and heme 515. **C** indicates the occurrence of two bonds of W (position 110); one with heme 515 and the other with heme 516. **D** indicates the absence of hydrogen bond with heme 515 at position 38 in the *Camponotus maculatus* barcode sequence due to substitution of tyrosine (Y) for phenyl alanine (F). As indicated in C, default position of W during 3D structure prediction is 126, while position 110 following protein model of Pentinsaari et al. (2016). Different types of atoms in the structure are indicated by different color.

**Fig 4:**
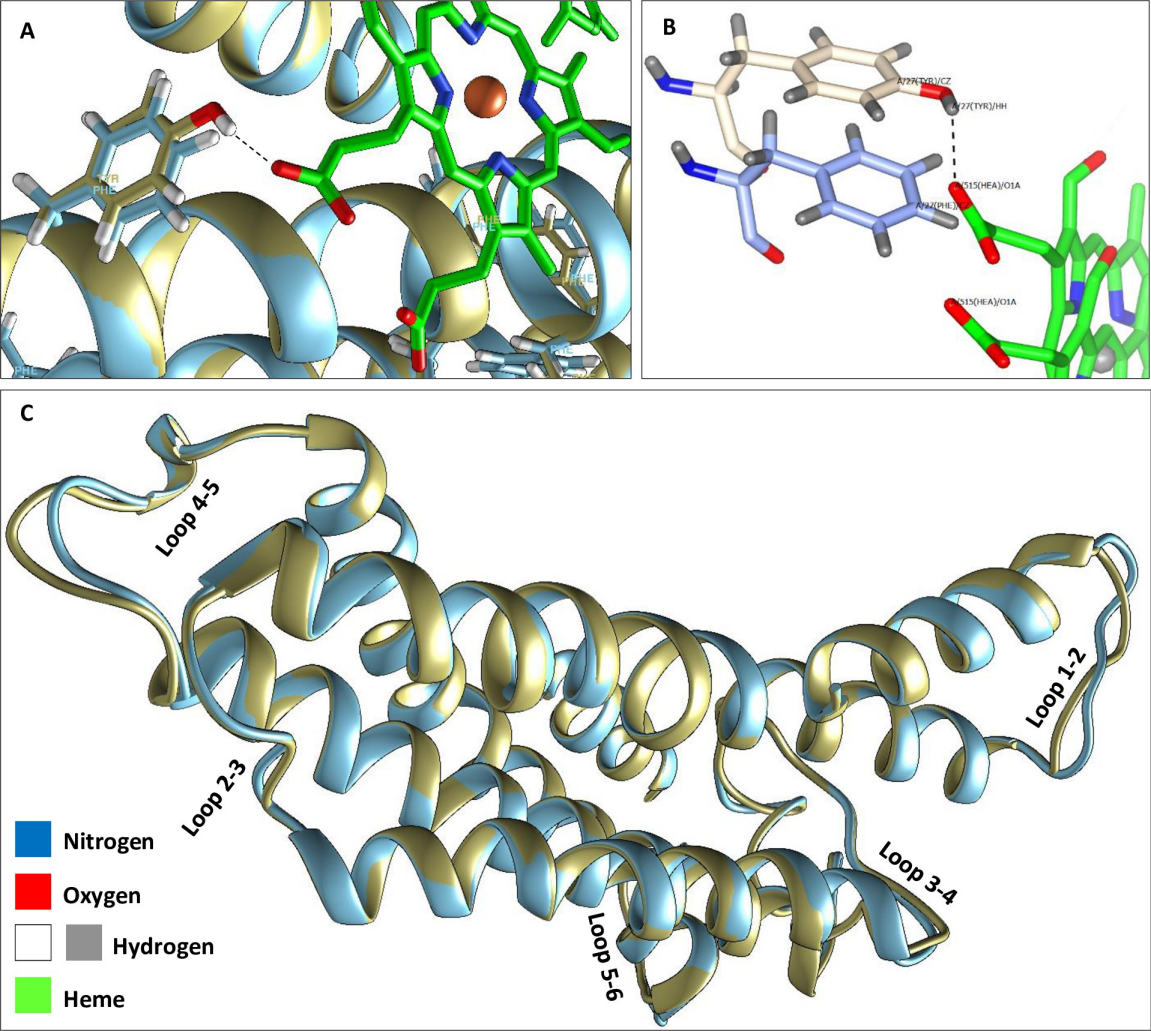
Alignment of the predicted three-dimensional structures of COI proteins of *Batrachedra amydraula* and *Camponotus maculatus*. **A** indicates the occurrence of a hydrogen bond between tyrosine of the first insect at position 38 and Heme 515, while lack of bonding between phenyl alanine of the second insect at the same position. **B** indicates the side chain structures of the two AAs facing Heme 515. **C** indicates the alignments of the six helices and five loops of the two 3D structures.

Functional constraints during the evolution of the insect COI barcode was checked in the predicted three-dimensional structure in terms of the distances between AAs and the two heme ligands referred to as hemes 515 and 516 (Fig 3). The analysis indicated a number of 16 AAs mostly in close proximity with heme 515 (Fig 4A). These AAs exist in helices 2 (10) and 1 (4) and loop 3–4 (2). They are Thr (T), Ser (S), Ile (I), Arg (R), Thy (Y), Ile (I), Val (V), His (H), Ala (A), Met (M), Ile (I), Met (M), Val (V), Ile (I), Gly (G) and Trp (W). They exist at codon positions 15, 18, 21, 22, 38, 41, 42, 45, 46, 49, 50, 53, 54, 57, 109 and 110, respectively. Six of them at positions 15, 21, 38, 41, 42 and 57 vary among the six insect orders, while the rest are conserved. We selected *Batrachedra amydraula* as a model barcode of the insect conserved sequences for the 16 AAs in the heme 515 vicinity in which the predicted 3D structure was generated (Fig 3). We mainly focused on four AAs that directly share six bonds with hemes 515 and 516. These AAs are R (position 22), Y (position 38), H (position 45) and W (position 110). Two bonds exist between H at position 45 and heme 515 (Fig 4B) and two other bonds exist between W and the two heme ligands 515 and 516 (Fig 3C). Chemical bonds occur between NH1 molecule of R (position 22) and OMA molecule of heme 515, while OH molecule of Y (position 38) and O1A molecule of heme 515. Two chemical bonds occur between NE2 molecule of H (position 45) side chain and both NA and NC molecules of heme 515. W (position 110) shares N atom of its backbone with O1D molecule of heme 515 and NE1 molecule of its side chain with O2D molecule of heme 516. Distances between the four selected AAs and heme ligands of COI barcode are shown in S6 Fig. All the four AAs are < 4 Å apart from the respective heme ligand.

S4 Fig for the AA multiple sequence alignment indicated that the four AAs are conserved among insect orders except for tyrosine (position 38) that unexpectedly showed substitution to phenylalanine in two Hymenopteran species, e.g, *Camponotus maculatus* and *Monomorium junodi*. These two AAs differ in the occurrence of an OH molecule at the side chain of tyrosine, which is lacking in phenylalanine. The predicted 3D structure of *Camponotus maculatus* was investigated at this position in order to detect the possible severity accompanying this substitution (Fig 3D). Tyrosine and phenylalanine share the same chemical group, however, the structure indicates no chemical bonding between the AA and heme 515 albeit the possible occurrence of Van der Waals interaction. This is the result of polarity change due to substitution that increases the chance of splitting heme from the protein. We speculate that this change in the 3D structure of COI and its consequence can impair the process of electron transfer from Cu to heme, thus, potentially affect energy metabolism in the mitochondria. Fig 3 depicts the alignment of the predicted three-dimensional structures of COI proteins of *Batrachedra amydraula* and *Camponotus maculatus*. The figure indicates the occurrence of a hydrogen bond between tyrosine of the first insect at position 38 and Heme 515 while lacking bond between phenylalanine of the second insect at the same position (Fig 4A and 4B). The figure also underpins poor alignment of loop 1–2 or 4–5 of the two insect species (Fig 3C), which reflects the high rate of varied AA in these two regions in line with the results of Pentinsaari et al. [32] and those of the present study (S4 Fig). The three-dimensional structure of the protein also indicates that the change from tyrosine to phenylalanine resulted in the loss of bonding with heme, thus likely affect energy metabolism. The phenylalanine appears to form a proper geometric complementarity; however, lacks electrostatic complementarity due to the absence of the hydroxyl group that contributes to the electrostatic interaction with the heme (Fig 4A), resulting in an increase in hydrophobicity. This suggests that the protein retains its structure, but not all of its functions. Moreover, the absence of a single hydrogen bond, computing the electrostatic potential showed no clear difference when tyrosine is substituted with phenylalanine (S5 Fig).

**Fig 5:**
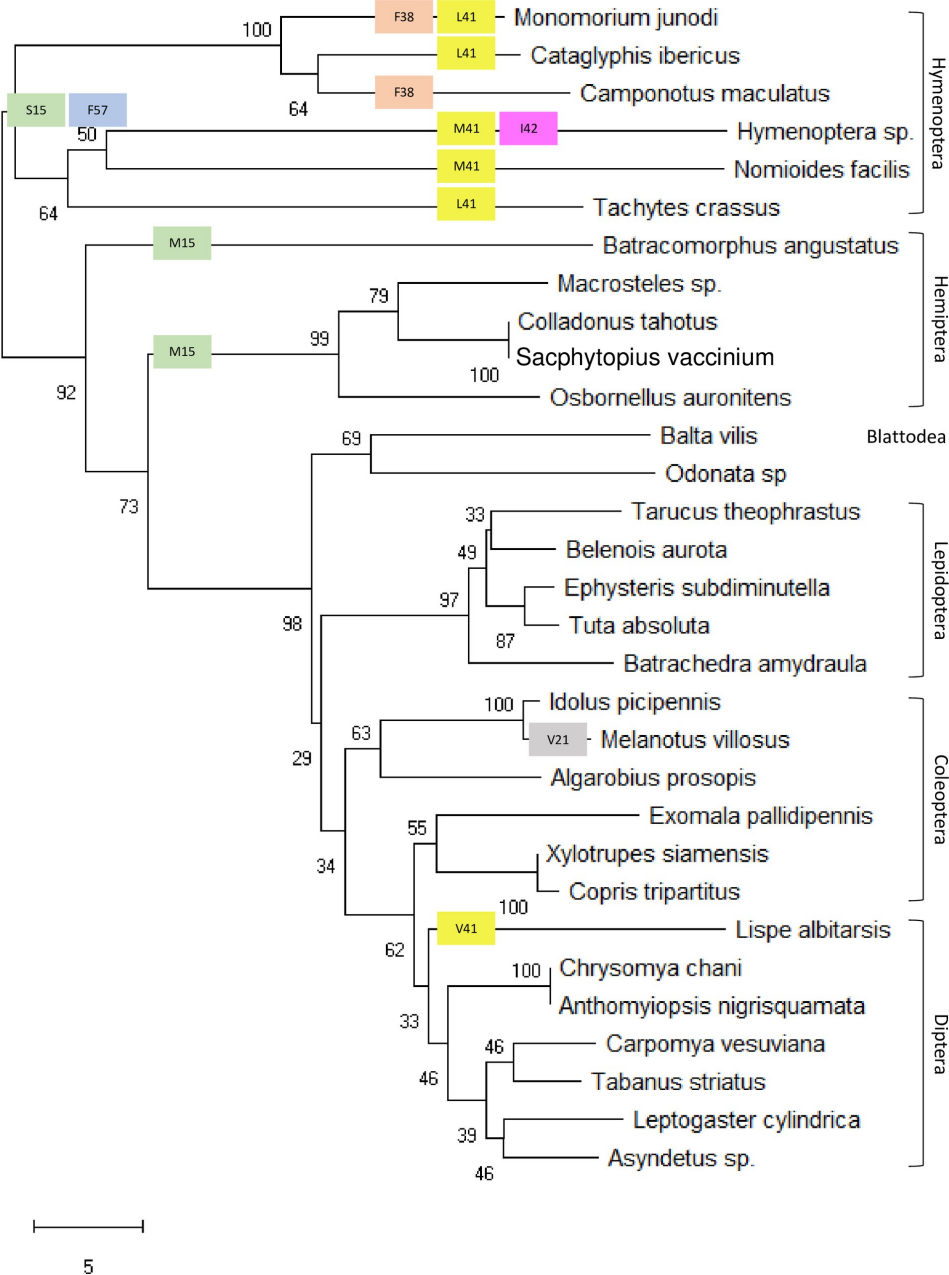
Phylogenetic tree of species of the six insect orders based on multiple AA sequence alignment in COI barcode. The tree also indicates substitutions of the six varied AAs (at positions 15, 21, 38, 41, 42 and 57) within the distance of < 4Å from heme 515. Different colors are given to substitutions at different positions, while same colors for substitutions at the same position. AA sequence of Odonata was used for comparison.

Phylogenetic tree based on multiple AA sequence alignment of COI barcode indicated that Hymenoptera is the most genetically distant of all the six insect orders under study (Fig 5). The tree structure complements that of the tree generated based on multiple DNA sequence alignment (S2 Fig) in which Blattodea was shown to be closest to Odonata. The same criterion applies to the relatedness between the two orders Hemiptera and Hymenoptera and the two other orders Coleoptera and Diptera. We further analyzed the six varied AAs at positions 15, 21, 38, 41, 42 and 57, existing within the distance of < 4Å from heme 515, in terms of transitions from one biochemical group to the other (Fig 5). At position 15 of the COI protein, threonine is changed to either serine with the same chemical group (polar uncharged) or methionine (two-codon AA) with different chemical groups (nonpolar) in all species of Hymenoptera and Hemiptera, respectively. At position 21, the substitution of isoleucine to valine in the same chemical group (nonpolar) has taken place in only *Melanotus villosus* species. At position 38, the substitution of tyrosine to phenylalanine in the same chemical group has taken place in the two Hymenopteran species *Monomorium junodi* and *Camponotus maculatus*. At position 41, the substitution of isoleucine (two-codon AA) to either leucine (six-codon AA) or methionine (two-codon AA) in the same chemical group (nonpolar) has taken place in Hymenopteran species except for *Camponotus maculatus*. An extra substitution at the same position has taken place to valine also in the same chemical group (nonpolar). At position 42, the substitution of valine to isoleucine in the same chemical group has taken place in Hymenopteran species, which was not fully detected at the species level. Interestingly, substitution at position 57 from isoleucine (nonpolar) to phenylalanine in a different chemical group (aromatic) has taken place in all Hymenopteran species. The AA transitions from one chemical group to the other might result in impaired energy metabolism. The study indicated a small distance between Hymenoptera and Hemiptera in the phylogenetic tree than between Coleoptera and Hemiptera. The data of AA substitution support our speculation that Hymenoptera is the most genetically distant of all the other insect orders under study. As Hymenopteran species are shown closely related to Hemipteran species and the fact that Hymenopteran species showed AA substitutions in five out of the six varied AAs in close proximity with the heme ligand indicate that Hymenoptera might be among the oldest insect orders.

**Fig 6:**
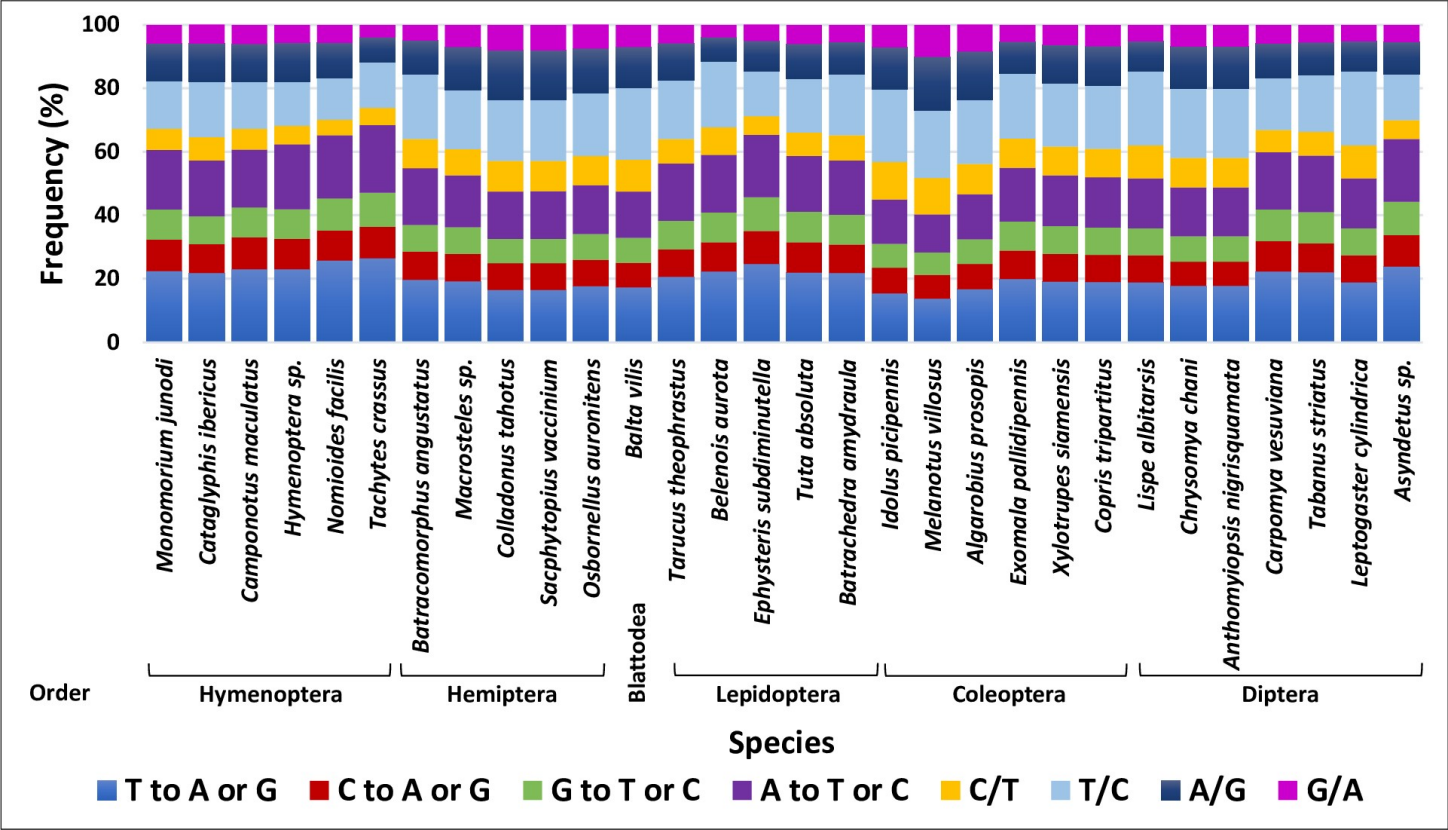
Nucleotide substitution matrix derived based on the COI barcode. Maximum composite likelihood estimates of the pattern of nucleotide substitution to detect transition and transversion frequencies based on DNA sequence alignment in COI barcode of species in the six insect orders.

The estimated nucleotide substitution matrices in the COI barcode of the six orders generally showed a slightly higher probability of transversion compared with transition (Fig 6). The probability of transversion ranged between ~40% for the Coleopteran species *Melanotus villosus* and ~65% for the Hymenopteran species *Tachytes crassus*. Substitutions of A followed by T to any other nucleotide represents the lowest probability compared with the rest. Changes from C to T followed by G to A showed the highest transition frequency compared with the others. These two high transition frequencies justify the high TA ratio in insect orders and the bias against G or C during evolution. Both Coleoptera and Diptera showed the most notable bias against G.

**Fig 7:**
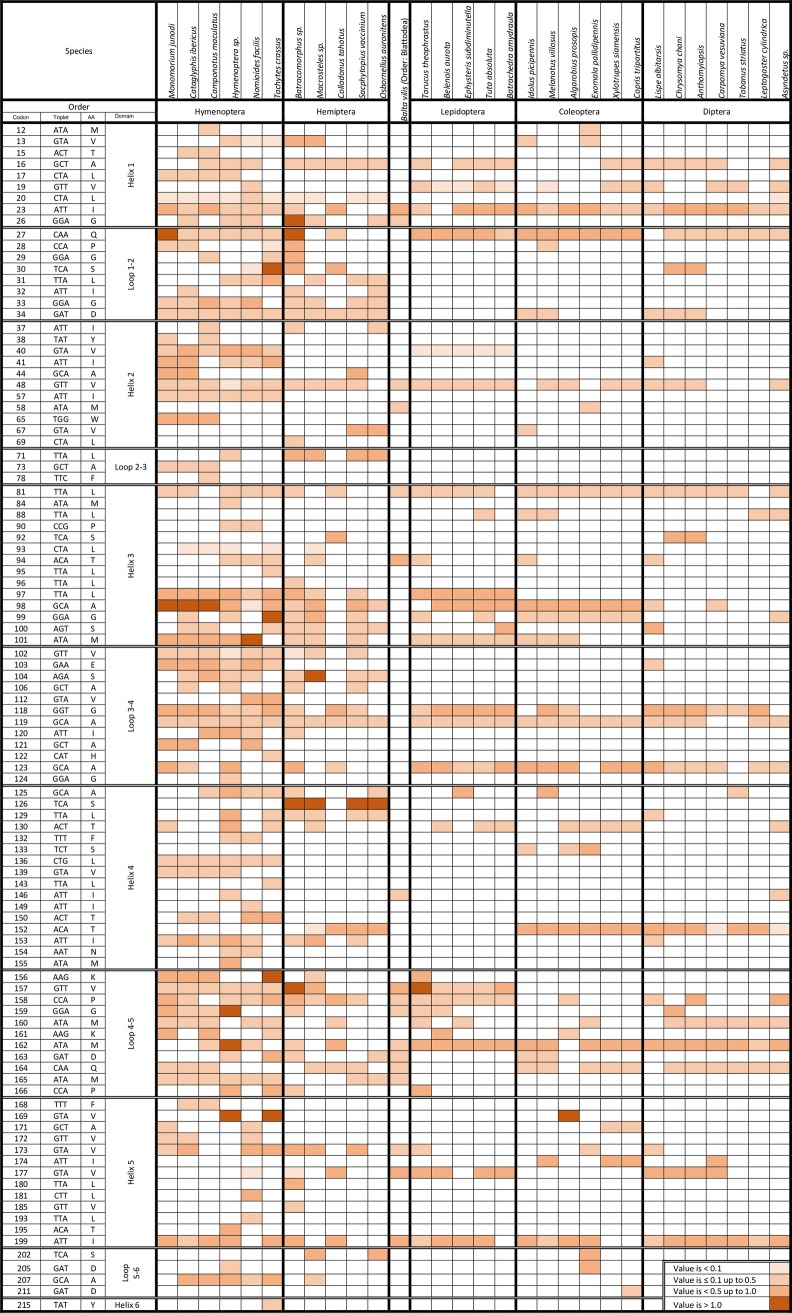
Maximum Likelihood computations of codon-by-codon synonymous (s) and nonsynonymous (n) substitutions in COI protein. Description of COI codons that are under positive selection based on the values of dN-dS in 30 species of six insect orders live in Saudi Arabia. Sequence of COI in order Odonata was used as a reference.

Maximum Likelihood computations of codon-by-codon synonymous (s) and nonsynonymous (n) substitutions in COI protein were conducted using the HyPhy software package in which Odonata was used as the reference ancestral order (Fig 7). The total number of nonsynonymous codons is 109, out of 211, of which 102 of them showed positive values at different rates across the six orders. The other seven codons showed negative dN-dS values, hence, listed under purifying selection. Hymenoperan followed by Hemipteran species are severely under positive selection as the values of dN-dS were positive in 90 and 61 out of the 102 codons, respectively. Twenty-two extremely high positive dN-dS values (> 1.0) in 13 codons were scored for species only of these two orders; the most frequent are codons 98 (A, a four-codon AA) for Hymenopteran species and 126 (S, an eight-codon AA) for Hemipteran species. On the other hand, the only species (e.g., *Balta vilis*) of Blattodea showed positive dN-dS values for as low as 17 codons. This low number reflects the close relatedness of this order with Odonata in line with the results of the phylogenetic analysis. The other three orders, e.g., Lepidoptera, Coleoptera, and Diptera showed a total of 30, 38 and 34 positive dN-dS values across species, respectively. This indicates that most codons of COI barcode in these three orders seem to be mostly under purifying selection. There are 16 unique codons to a given species of either Hymenopteran (13 codons) or Hemipteran (3 codons) that are under positive selection. These Hymenopteran species are *Camponotus maculatus* (1 codon), *Hymenoptera* sp. (4 codons), *Nomioides facilis* (3 codons) and *Tachytes crassus* (4 codons), while Hemipteran species is *Batracomorphus angustatus* (4 codons). On the other hand, 10 codons frequently showed positive selection across most species of the six orders; five of them are located in the helix regions and five are located in the loop regions. Positions of these codons are 16, 23, 27, 48, 81, 118, 119, 123, 162 and 199. As for the dN-dS values in terms of domain structure, the results indicated that percentages of codons under positive selection are ~ 80, 31, 61, 52, 40 and 9% in helices 1 to 6, while 100, 38, 52, 100 and 33% in loops 1–2 to 5–6, respectively. These results indicate that codons in helices 2, 5 and 6 and in loops 2–3 and 5–6 are mostly conserved and are under strong purifying selection.

The study indicates that COI barcodes can provide insights by which the encoded protein evolve and function in insect at different taxonomic scales. The COI or Folmer region is basically used for species identification based on variations at the DNA level [34]. The region is sufficiently conserved within species, while variable among species. This region is located in the enzymatically active part of COI, thus involved in the respiratory chain by transferring electrons from Cu to heme. Accordingly, this region is under functional constraints and mutations in close proximity with heme ought to be lethal. There is less evolutionary pressure against variation in positions within the extra-membranous loop structures (e.g., loops 1–2 at AA positions 27–34, 3–4 at AA positions 102–124 and 4–5 at AA positions 156–166). This conclusion was previously reached by Panchenko et al. [35]. Amino acids in the two highly conserved loops (e.g., loops 2–3 and 5–6) seem important in holding the two sides of the enzyme and avoid severe conformational changes. However, tryptophan within the variable loop 3–4 at position 99 rigidly evolves as it is the only AA of COI protein that bonds with the two hemes. Another AA in positions close to the active site is rigid membrane-embedded α-helices undergo functional constraints and restricted evolution (negative evolution). A similar pattern of variation was observed in the protein encoded by mitochondrial ribosomal RNA gene, where the loops freely evolve [36].

The study generally indicated that almost half the positions of the AA of the barcode region are variable, however, they are mostly beyond the atomic interaction distance of the heme groups as previously reported [27,16,37]. Six of these AAs exist within a distance of >4Å with four of them changed to AAs of the same chemical groups. However, the three-dimensional structure of the protein indicates that the change from tyrosine to phenylalanine resulted in the loss of bonding with heme, thus likely affect energy metabolism.

Estimates of dN-dS were used for detecting codons under either positive or purifying selection. dS is the number of synonymous substitutions per site (s/S) and dN is the number of nonsynonymous substitutions per site (n/N). Positive values indicate the overabundance of nonsynonymous substitutions in these positions. During evolution, positive (Darwinian) selection promotes the sweeps of new beneficial alleles, and negative (or purifying) selection impedes the spread of harmful alleles [38]. The results indicated that 102 codons are under positive selection and tend to evolve more rapidly.

Phenotypic plasticity or polyphenism is another potential player of evolutionary changes and shaping of ecosystems that allows species to phenotypically adapt to different environmental conditions during evolution [39,40,41,42]. This type of evolutionary force is not genome structure-based, rather, it relies on the differential gene expression (gene expression-biased) and is particularly prominent in insects. Gene expression bias is predominant in molecular evolution when selection becomes less effective at removing deleterious alleles. Studying polyphenism provides insight into the molecular basis of phenotypic differentiation during evolution [43,44,45,46, 47,48,49,50,51]. This approach is ideal especially for cases where low genetic differences exist among species [52,53].

Hymenopteran species have the highest AA variation in line with the presumed age of the order that is likely among the oldest, thus had a long time to molecularly evolve by changing AA sequence. Previous reports speculated that differences in patterns of variation, substitution, and selection among insect orders are due to the number of metabolism-related factors that are high in actively flying insect species (like Hymenoptera) than in non-flying species [54]. Selection is biased towards the higher active metabolic rate and the increase of the resting metabolic rate. Pentinsaari et al. [32] indicated a higher relative rate of CT/GA transitions in the insect is the result of higher oxidative damage to DNA. This high variation is the result of a weak purifying selection in Hymenopteran species. We strongly recommend making wet laboratory analysis to support whether the changes in AAs consequently affect metabolism in the target species or not. The overall results argue the possible evolutionary position of Hymenopteran species among those of other insect orders living in Saudi Arabia, especially Hemiptera and Coleoptera.

## Supporting information

S1 Fig Photographs of the insect species collected for the study. Photographs of specimen representing the 30 insect species collected from Hada Al-Sham station, KAU, Saudi Arabia.
(TIF)

S2 Fig Multiple sequence alignment of the COI DNA barcodes. A comparative nucleotide sequence analysis of 634 bp that formed the specie specific insect barcode along with a species from Odonata used as a reference.
(TIF)

S3 Fig Phylogenetic tree analysis of the insect orders. Phylogenetic tree describing the genetic relatedness among species of the six orders based on DNA sequences of the COI gene fragment. Consensus DNA sequence of Odonata was used for comparison.
(TIF)

S4 Fig Multiple sequence alignment of the COI protein sequences. A comparative aminoacid sequence alignment of protein fragment spanning 211 AA of the 30 insect species along with a reference sequence from Odonata for comparison. AA sequences start at position 12 and ends at position 222 following the numbering of Pentinsaari et al. (2016).
(TIF)

S5 Fig **Computation calculation of the electrostatic potential of COI protein in the presence of Tyr (A) or Phe (B).** Positive potential is shown in blue, while the negative potential is shown in red at pH7. Diagrams were generated using DeepView-Swiss-PdbViewer (v4.1) (http://www.expasy.org/spdbv).
(TIF)

S6 Fig Description of the predicted three-dimensional model pf the COI protein structure. Predicted three-dimensional model of the COI protein structure of *Batrachedra amydraula* indicating the bond distances (indicated by black arrows) between the two heme structures 515 and 516 in one hand and AAs arginine (R), tyrosine (Y), histidine (H) and tryptophan (W) on the other hand. The four AAs exist in positions 11, 27, 34 and 99 of the COI polypeptide chain, respectively. **A** displays bonds of heme/R (one bond), heme/Y (one bond) and heme/H (two bonds). The latter three AAs bond with heme 515. **B** displays bonds of heme/W (two bonds) to indicate that W is the only AA of the COI that bonds (once) with heme 516. **C** displays the entire six bonds of the four AAs and the two hemes. The distance between any given AA and heme 515 or between W and heme 516 is <4Å. Different types of atoms in the structure are indicated by a different color.
(TIF)

## References

[1] Scheffers BR, Joppa LN, Pimm SL, Laurance WF. What we know and don’t know about Earth's missing biodiversity. Trends Ecol. Evol. 2012;27:501–510. 10.1016/j.tree.2012.05.008 22784409

[2] Hebert PDN, Cywinska A, Ball SL, deWaard JR. Biological identifications through DNA barcodes. Proc. Biol. Sci. 2003;270:313–321. 10.1098/rspb.2002.2218 12614582 PMC1691236

[3] Hebert PDN, Penton EH, Burns JM, Janzen DH, Hallwachs W. Ten species in one: DNA barcoding reveals cryptic species in the neotropical skipper butterfly Astraptes fulgerator. Proc. Natl. Acad. Sci. 2004;101:14812–14817. 10.1073/pnas.0406166101 15465915 PMC522015

[4] Ratnasingham S and Hebert PDN. A DNA-based registry for all animal species: The Barcode Index Number (BIN) System. PLoS ONE. 2013;8: e66213 10.1371/journal.pone.0066213 23861743 PMC3704603

[5] Hebert PDN, deWaard JR, Zakharov EV, Prosser SWJ, Sones JE, et al A DNA “Barcode Blitz”: rapid digitization and sequencing of a natural history collection. PLoS ONE. 2013; 8:e68535 10.1371/journal.pone.0068535 23874660 PMC3707885

[6] Hebert PDN, Ratnasingham S, Zakharov EV, Telfer AC, Levesque-Beaudin V, Milton MA, et al Counting species with DNA barcodes: Canadian insects. Phil. Trans. R. Soc. 2016;B371:20150333. 10.1098/rstb.2015.0333 PMC4971185 27481785

[7] Cristescu ME. From barcoding single individuals to metabarcoding biological communities: towards an integrative approach to the study of global biodiversity. Trends Ecol. Evol. 2014;29:566–571. 10.1016/j.tree.2014.08.001 25175416

[8] Gwiazdowski RA, Foottit RG, Maw HEL, Hebert PDN. The Hemiptera (Insecta) of Canada: Constructing a reference library of DNA barcodes. PLoS ONE. 2015;10:e0125635 10.1371/journal.pone.0125635 25923328 PMC4414572

[9] Mutanen M, Kekkonen M, Prosser SWJ, Hebert PDN, Kaila L. One species in eight: DNA barcodes from type specimens resolve a taxonomic quagmire. Mol. Ecol. Resour. 2015;15:967–984. 10.1111/1755-0998.12361 25524367 PMC4964951

[10] Borges LMS, Hollatz C, Lobo J, Cunha AM, Vilela AP, Calado G, et al With a little help from DNA barcoding: investigating the diversity of Gastropoda from the Portuguese coast. Sci. Rep. 2016;6:20226 10.1038/srep20226 26876495 PMC4753432

[11] Ashfaq M and Hebert PDN. DNA barcodes for bio-surveillance: regulated and economically important arthropod plant pests. Genome. 2016;59:933–945. 10.1139/gen-2016-0024 27753511

[12] Ren JM, Ashfaq M, Hu XN, Ma J, Liang F, Hebert PDN, et al Barcode index numbers expedite quarantine inspections and aid the interception of nonindigenous mealybugs (Pseudococcidae). Biol. Invasions. 2018;20:449–460.

[13] Ashfaq M, Sabir JSM, El-Ansary HO, Perez K, Levesque-Beaudin V, Khan AM, et al Insect diversity in the Saharo-Arabian region: Revealing a little-studied fauna by DNA barcoding. PLoS ONE. 2018;13:e0199965 10.1371/journal.pone.0199965 29985924 PMC6037371

[14] Steinke D, Breton V, Berzitis E, Hebert P D N. The School Malaise Trap Program: Coupling educational outreach with scientific discovery. PLoS Biol. 2017;15:e2001829 10.1371/journal.pbio.2001829 28437475 PMC5402927

[15] Moriniere J, de Araujo BC, Lam AW, Hausmann A, Balke M, Schmidt S, et al Species identification in Malaise trap samples by DNA barcoding based on NGS technologies and a scoring matrix. PLoS ONE. 2016;11:e0155497 10.1371/journal.pone.0155497 27191722 PMC4871420

[16] Tsukihara T, Aoyama H, Yamashita E, Tomizaki T, Yamaguchi H, Shinzawa-Itoh K, et al The whole structure of the 13-subunit oxidized cytochrome c oxidase at 2.8 Å. Science. 1996;272:1136–1144. 10.1126/science.272.5265.1136 8638158

[17] Balsa E, Marco R, Perales-Clemente E, Szklarczyk R, Calvo E, Landázuri MO, et al NDUFA4 is a subunit of Complex IV of the mammalian electron transport chain. Cell Metab. 2012;16:378–386. 10.1016/j.cmet.2012.07.015 22902835

[18] Mathews CK, Holde KE. van, Appling DRand Anthony-Cahill SJ, 2013 Biochemistry 4th edition (Pearson).

[19] Pesole G, Gissi C, De Chirico A, Saccone C. Nucleotide substitution rate of mammalian mitochondrial genomes. J. Mol. Evol. 1999;48:427–434. 10.1007/pl00006487 10079281

[20] Meiklejohn CD, Montooth KL, Rand DM. Positive and negative selection on the mitochondrial genome. Trends Genet. 2007;23:259–263. 10.1016/j.tig.2007.03.008 17418445

[21] Castoe TA, Jiang ZJ, Gu W, Wang ZO, Pollock DD. Adaptive evolution and functional redesign of core metabolic proteins in snakes. PLoS ONE. 2008;3:e2201 10.1371/journal.pone.0002201 18493604 PMC2376058

[22] Galtier N, Nabholz B, Glémin S, Hurst GD. Mitochondrial DNA as a marker of molecular diversity: A reappraisal. Mol. Ecol. 2009;18:4541–4550. 10.1111/j.1365-294X.2009.04380.x 19821901

[23] Hutcheson J and Jones D. Spatial variability of insect communities in a homogenous system: Measuring biodiversity using Malaise trapped beetles in a Pinus radiata plantation in New Zealand. Forest Ecol. Manag. 1999;118:93–105.

[24] Hill D, Fasham M, Tucker G, Shewry M, Shaw P. Handbook of biodiversity methods: Survey, evaluation and monitoring Cambridge University Press 2005;573p.

[25] Evans N, Paulay G. DNA barcoding methods for invertebrates. Meth. Mol. Biol. 2012;858:47–77. 10.1007/978-1-61779-591-6_4 22684952

[26] Bybee S, Córdoba-Aguilar A, Catherine DM, Futahashi R, Hansson B. Odonata (dragonflies and damselflies) as a bridge between ecology and evolutionary genomics. Front. in Zoology. 2016;13:46. 10.1186/s12983-016-0176-7 PMC5057408 27766110

[27] Tsukihara T, Aoyama H, Yamashita E, Tomizaki T, Yamaguchi H, et al Structures of metal sites of oxidized bovine heart cytochrome c oxidase at 2.8 Å. Science. 1995;269:1069–1074. 10.1126/science.7652554 7652554

[28] Yang J, Yan R, Roy A, Xu D, Poisson J. The I-TASSER Suite: Protein structure and function prediction. Nature Methods. 2015;12:7–8. 10.1038/nmeth.3213 25549265 PMC4428668

[29] Pettersen EF, Goddard TD, Huang CC, Couch GS, Greenblatt DM, Meng EC, et al UCSF Chimera—a visualization system for exploratory research and analysis. J. Comput. Chem. 2004;25:1605–1612. 10.1002/jcc.20084 15264254

[30] Muse SV and Gaut BS. A likelihood approach for comparing synonymous and nonsynonymous nucleotide substitution rates, with application to the chloroplast genome. Mol. Biol. Evol. 1994;11:715–724. 10.1093/oxfordjournals.molbev.a040152 7968485

[31] Felsenstein J. Evolutionary trees from DNA sequences: a maximum likelihood approach. J. Mol. Evol. 1981;17:368–376. 10.1007/bf01734359 7288891

[32] Pentinsaari M, Salmela H, Mutanen M, Roslin T. Molecular evolution of a widely adopted taxonomic marker (COI) across the animal tree of life. Sci. Rep. 2016;6:35275 10.1038/srep35275 27734964 PMC5062346

[33] Córdoba-Aguilar A. Dragonflies and damselflies. Model organisms for ecological and evolutionary research Oxford: Oxford University Press 2008.

[34] Folmer O, Black M, Hoeh W, Lutz R, Vrijenhoek R. DNA primers for amplification of mitochondrial cytochrome c oxidase subunit I from diverse metazoan invertebrates. Mol. Marine Biol. Biotechnol. 1994:3;294–299. 7881515

[35] Panchenko AR, Wolf YI, Panchenko LA, Madej T. Evolutionary plasticity of protein families: Coupling between sequence and structure variation. Proteins. 2005;61:535–544. 10.1002/prot.20644 16184609 PMC1941674

[36] Ortí G, Petry P, Porto JIR, Jégu M, Meyer A. Patterns of nucleotide change in mitochondrial ribosomal RNA genes and the phylogeny of piranhas. J. Mol. Evol. 1996;42:169–182. 10.1007/bf02198843 8919869

[37] Tsukihara T, Shimokata K, Katayama Y, Shimada H, Muramoto K, Aoyama H, et al The low-spin heme of cytochrome c oxidase as the driving element of the proton-pumping process. Proc. Natl. Acad. Sci. 2003;100:15304–15309. 10.1073/pnas.2635097100 14673090 PMC307562

[38] Page RDM, Holmes EC. Molecular Evolution: A Phylogenetic Approach (Blackwell Science, 1998 Oxford). 10.1126/science.280.5367.1256

[39] Moran NA. The evolutionary maintenance of alternative phenotypes. Am. Nat. 1992;139:971–989.

[40] Nijhout HF. Development and evolution of adaptive polyphenisms. Evol. Dev. 2003;5:9–18. 12492404 10.1046/j.1525-142x.2003.03003.x

[41] West-Eberhard MJ. Developmental plasticity and evolution 2003 Oxford Univ Press, New York.

[42] Pfennig DW, Wund MA, Snell-Rood EC, Cruickshank T, Schlichting CD, Moczwk AP. Phenotypic plasticity's impacts on diversification and speciation. Trends Ecol. Evol. 2010;25:459–467. 10.1016/j.tree.2010.05.006 20557976

[43] Hölldobler B, Wilson EO. The Ants 1990 Belknap Press of Harvard Univ. Press, Cambridge, MA.

[44] Moczek AP and Emlen DJ. Proximate determination of male horn dimorphism in the beetle Onthophagus Taurus (Coleoptera: Scarabaeidae). J. Evol. Biol. 1999;12:27–37.

[45] Müller CB, Williams IS, Hardie J. The role of nutrition, crowding and interspecific interactions in the development of winged aphids. Ecol. Entomol. 2001;26:330–340.

[46] Ellegren H and Parsch J. The evolution of sex-biased genes and sex-biased gene expression. Nat. Rev. Genet. 2007;8:689–698. 10.1038/nrg2167 17680007

[47] Smith CR, Toth AL, Suarez AV, Robinson GE. Genetic and genomic analyses of the division of labour in insect societies. Nat. Rev. Genet. 2008;9:735–748. 10.1038/nrg2429 18802413

[48] Ayroles JF, Carbone MA, Stone EA, Jordan KW, Lyman RF, Magwire MM, et al Systems genetics of complex traits in Drosophila melanogaster. Nat. Genet.2009;41:299–307. 10.1038/ng.332 19234471 PMC2752214

[49] Connallon T and Clark AG. Association between sex-biased gene expression and mutations with sex-specific phenotypic consequences in Drosophila. Genome Biol. Evol. 2011;3:151–155. 10.1093/gbe/evr004 21292631 PMC3048362

[50] Graveley BR, Brooks AN, Joseph CW, Duff MO, Jane M, et al The developmental transcriptome of Drosophila melanogaster. Nature. 2011;471:473–479. 10.1038/nature09715 21179090 PMC3075879

[51] Ometto L, Shoemaker D, Ross KG, Keller L. Evolution of gene expression in fire ants: The effects of developmental stage, caste, and species. Mol. Biol. Evol. 2011;28:1381–1392. 10.1093/molbev/msq322 21172833

[52] Hunt BG, Wyder S, Elango N, Werren JH, Zdobnov EM, et al Sociality is linked to rates of protein evolution in a highly social insect. Mol. Biol. Evol. 2010;27:497–500. 10.1093/molbev/msp225 20110264

[53] Snell-Rood EC, Cash A, Han MV, Kijimoto T, Andrews J, Moczek AP. Developmental decoupling of alternative phenotypes: Insights from the transcriptomes of horn-polyphenic beetles. Evolution. 2011;65:231–245. 10.1111/j.1558-5646.2010.01106.x 20731717 PMC3010270

[54] Reinhold K. Energetically costly behaviour and the evolution of resting metabolic rate in insects. Funct. Ecol. 1999;13:217–224.

