# Supplementary figures and images for: Molecular evolution of cytochrome C oxidase-I protein of insects living in Saudi Arabia
Source: PLoS One. 2019 Nov 4;14(11):e0224336. doi: 10.1371/journal.pone.0224336 (PMC6827904; doi:10.1371/journal.pone.0224336)

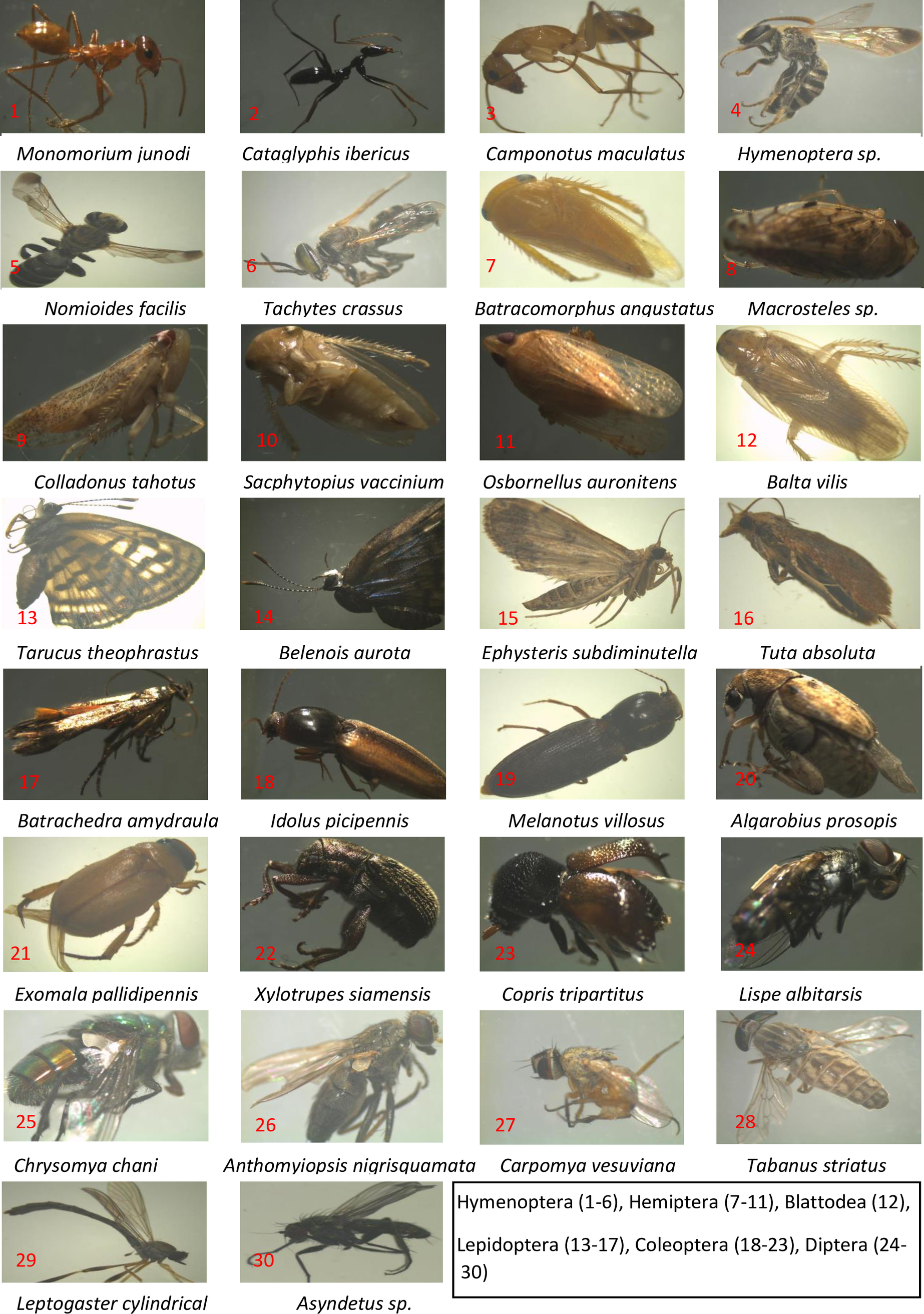

Supplement: S1 Fig — Photographs of specimen representing the 30 insect species collected from Hada Al-Sham station, KAU, Saudi Arabia. (TIF) [file pone.0224336.s001.tif]

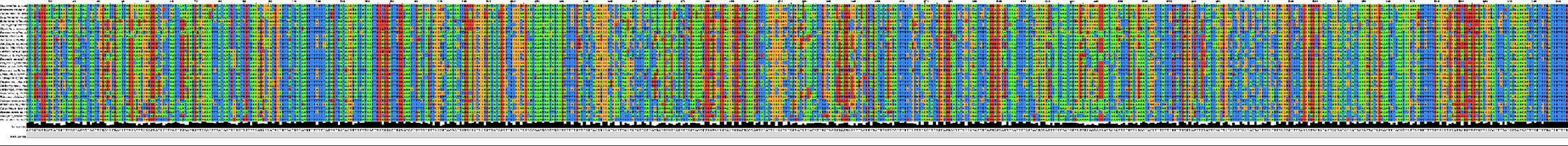

Supplement: S2 Fig — A comparative nucleotide sequence analysis of 634 bp that formed the specie specific insect barcode along with a species from Odonata used as a reference. (TIF) [file pone.0224336.s002.tif]

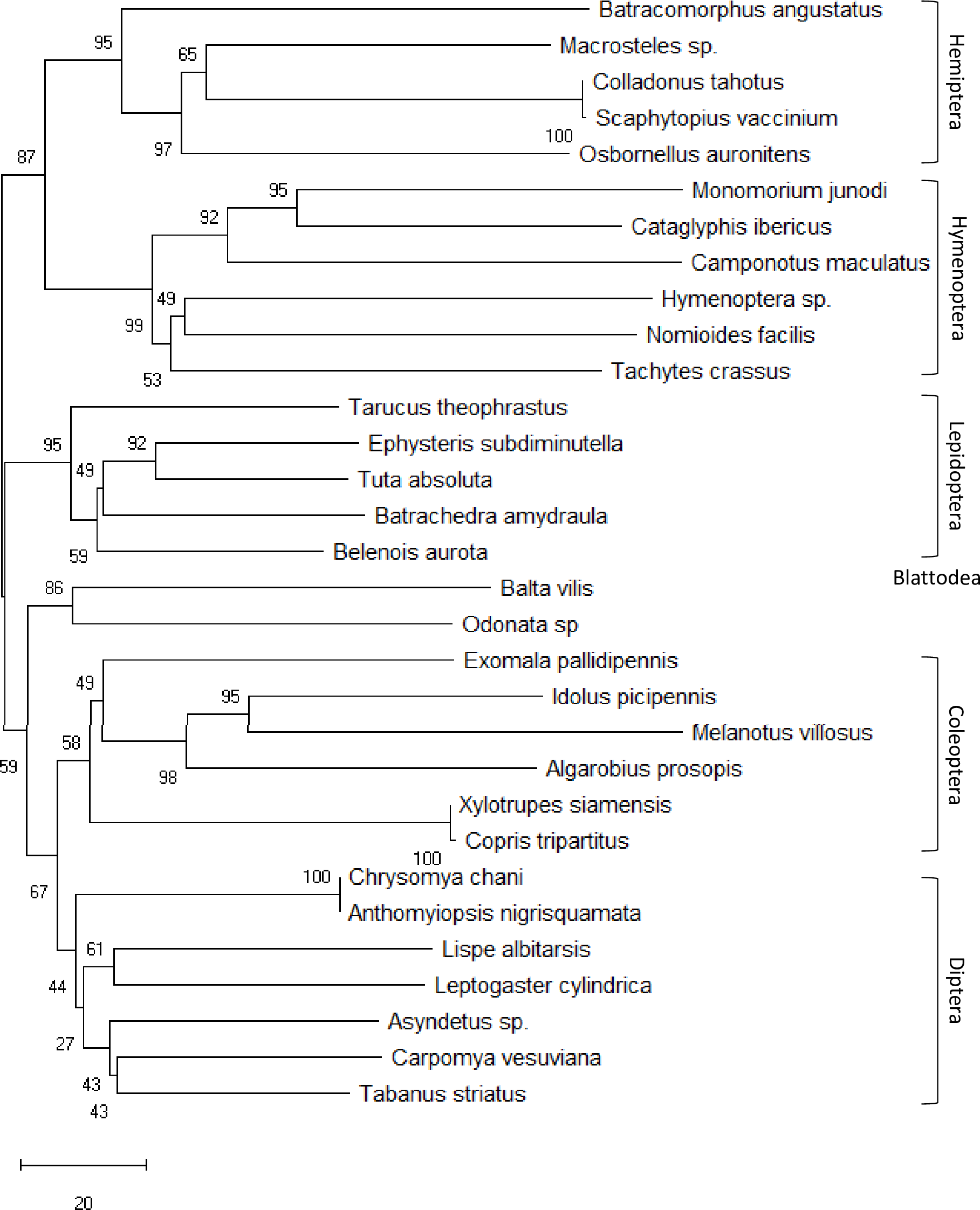

Supplement: S3 Fig — Phylogenetic tree describing the genetic relatedness among species of the six orders based on DNA sequences of the COI gene fragment. Consensus DNA sequence of Odonata was used for comparison. (TIF) [file pone.0224336.s003.tif]

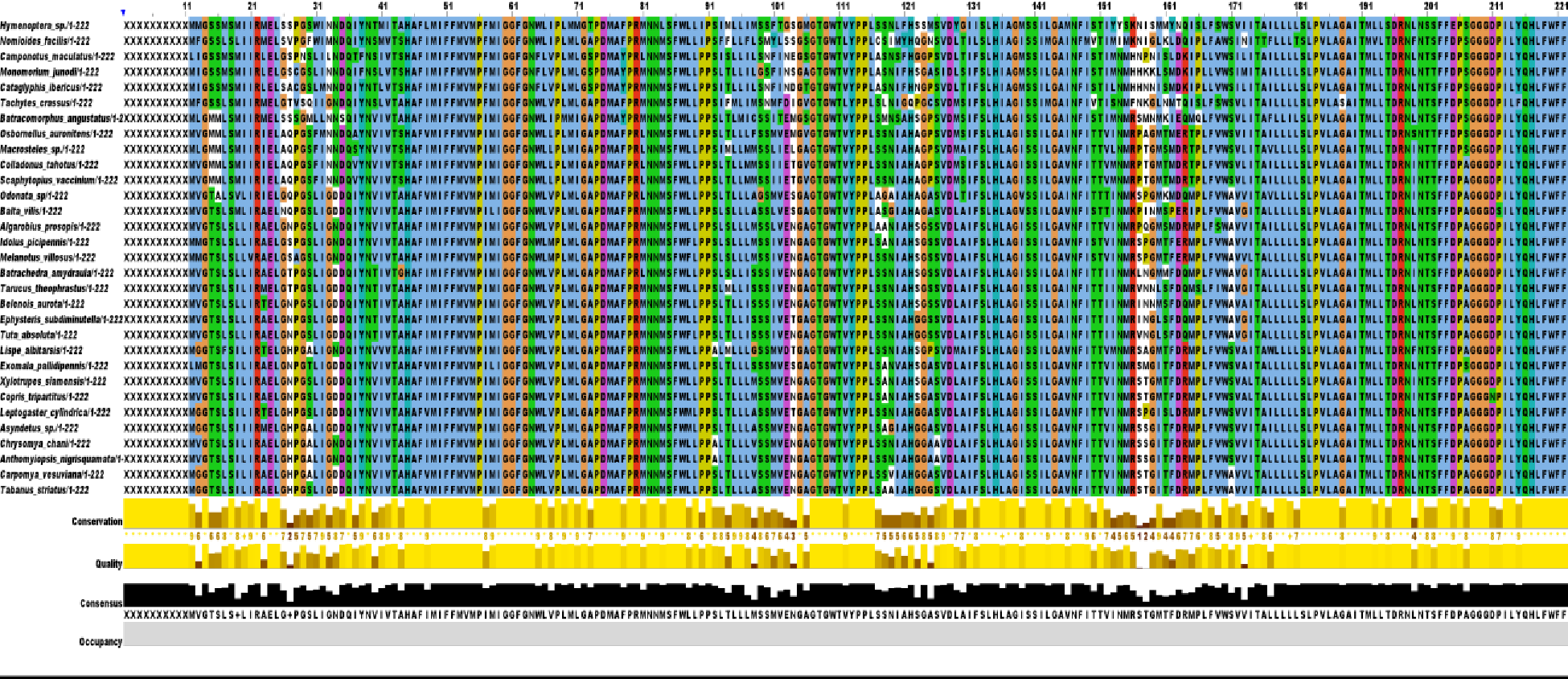

Supplement: S4 Fig — A comparative aminoacid sequence alignment of protein fragment spanning 211 AA of the 30 insect species along with a reference sequence from Odonata for comparison. AA sequences start at position 12 and ends at position 222 following the numbering of Pentinsaari et al. (2016). (TIF) [file pone.0224336.s004.tif]

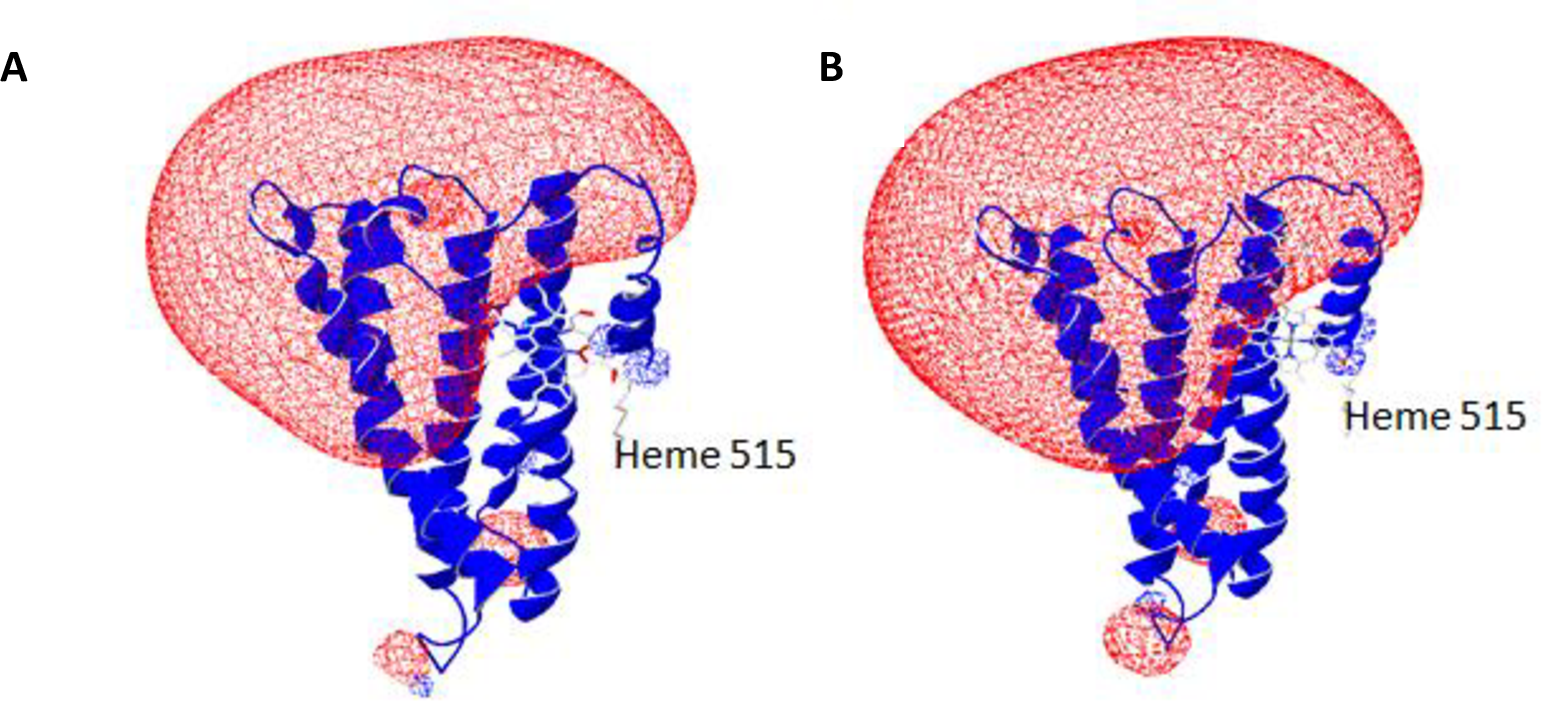

Supplement: S5 Fig — Computation calculation of the electrostatic potential of COI protein in the presence of Tyr (A) or Phe (B). Positive potential is shown in blue, while the negative potential is shown in red at pH7. Diagrams were generated using DeepView-Swiss-PdbViewer (v4.1) (http://www.expasy.org/spdbv). (TIF) [file pone.0224336.s005.tif]

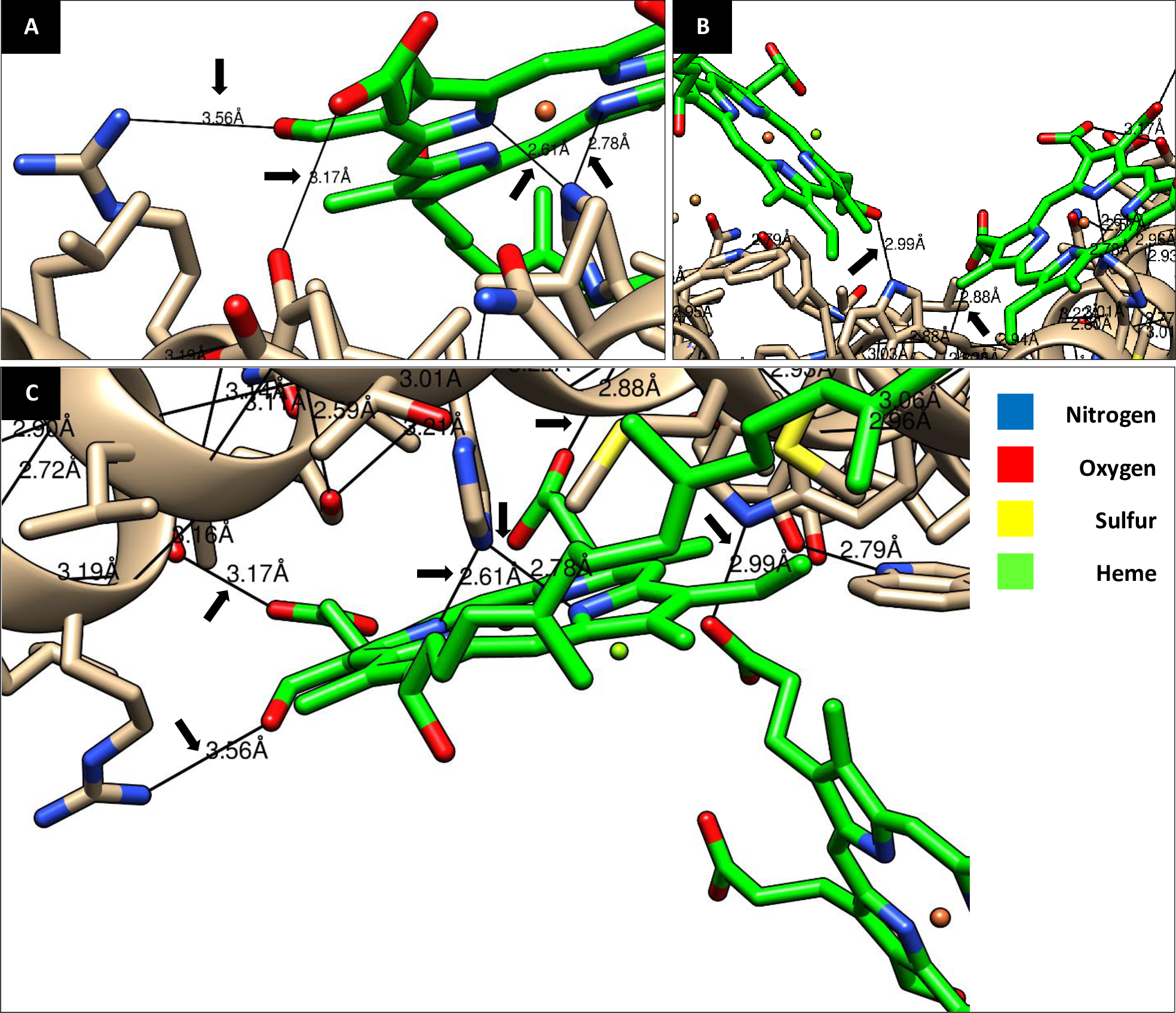

Supplement: S6 Fig — Predicted three-dimensional model of the COI protein structure of Batrachedra amydraula indicating the bond distances (indicated by black arrows) between the two heme structures 515 and 516 in one hand and AAs arginine (R), tyrosine (Y), histidine (H) and tryptophan (W) on the other hand. The four AAs exist in positions 11, 27, 34 and 99 of the COI polypeptide chain, respectively. A displays bonds of heme/R (one bond), heme/Y (one bond) and heme/H (two bonds). The latter three AAs bond with heme 515. B displays bonds of heme/W (two bonds) to indicate that W is the only AA of the COI that bonds (once) with heme 516. C displays the entire six bonds of the four AAs and the two hemes. The distance between any given AA and heme 515 or between W and heme 516 is <4Å. Different types of atoms in the structure are indicated by a different color. (TIF) [file pone.0224336.s006.tif]
